# Age Differences in Patterns of Cannabis Use Among U.S. Adults Who Consume Cannabis Frequently

**DOI:** 10.1093/geroni/igaf122.1971

**Published:** 2025-12-31

**Authors:** Ofir Livne, Alan Budney, Jacob Borodovsky, Deborah Hasin

**Affiliations:** Columbia University Irving Medical Center, New York, New York, United States; Dartmouth College, Hanover, New Hampshire, United States; Dartmouth College, Hanover, New Hampshire, United States; Columbia University, New York, New York, United States

## Abstract

Cannabis use has risen disproportionately among middle-aged and older U.S. adults, groups vulnerable to adverse effects, including cannabis use disorder (CUD). While consumption patterns have diversified, age-related differences in such patterns remain understudied. This study examined variations in cannabis behaviors and the relationship between quantity—measured in milligrams of THC (mgTHC)—and CUD in regular users. A total of 4,134 U.S. adults who reported daily cannabis use completed an online survey assessing consumption patterns and DSM-5 CUD criteria. Comparisons across sex, reasons for use, methods of consumption, CUD severity, criteria count, and mgTHC were conducted across three age groups (18–49, 50–64, 65+). Regression models, adjusted for sex and reasons for use, analyzed age-specific associations between mgTHC and CUD. Over 70% of participants used cannabis for both medical and recreational purposes. Middle-aged adults reported medical use more frequently than younger (18.1% vs. 13.7%; p < 0.001) and older adults (14.1%; p = 0.027), while older adults more often reported recreational-only use than middle-aged adults (15.8% vs. 10.5%; p = 0.002). Smoking buds was the most common method, with high-potency concentrate use declining with age. Daily mgTHC was positively associated with CUD severity, though middle-aged and older adults endorsed fewer CUD criteria than younger users at all mgTHC levels, with no significant age effects. Findings highlight age-related differences in consumption and reasons for use. Despite consuming less than younger users, older adults remain at risk, underscoring the need for targeted prevention strategies.

